# The role and application potential of cytochrome c in breast cancer therapy

**DOI:** 10.1007/s12672-025-03822-3

**Published:** 2025-11-05

**Authors:** Hongshuang Yue, Yumei Fu, Xiuyue Fan, Fulin Zhou, Shu Liu

**Affiliations:** 1 Center of Breast Diseases, Affiliated Hospital of Guizhou Medical University, 28 Guiyi Street, Guiyang, 550004 Guizhou China; 2 Department of Clinical Medicine, Guizhou Medical University, Guiyang, 550004 China; 3 Department of Breast Surgery, GuiYang Maternal and Child Health Care Hospital, Guiyang, 550004 China

**Keywords:** Cytochrome c, Apoptosis, Breast cancer

## Abstract

Apoptosis plays an important role in all stages of normal breast development, including duct morphogenesis, lactation and proliferative regression. Abnormal apoptosis of breast cells leads to the occurrence of tumors. Studies have shown that the status of cytochrome c (cyt c) in the mitochondrial apoptotic pathway is crucial to the occurrence of cancer and is at the core of this pathway. The response of tumors to treatments such as chemotherapy, radiotherapy and endocrine therapy is, to a certain extent, achieved by inducing the release of cyt c and triggering the intrinsic pathway of apoptosis. cyt c has potential value in cancer treatment. The purpose of this review is to review the relevant literature, systematically explore the role of cyt c in the pathogenesis and treatment of breast cancer.

## Introduction

**Fig. 1:**
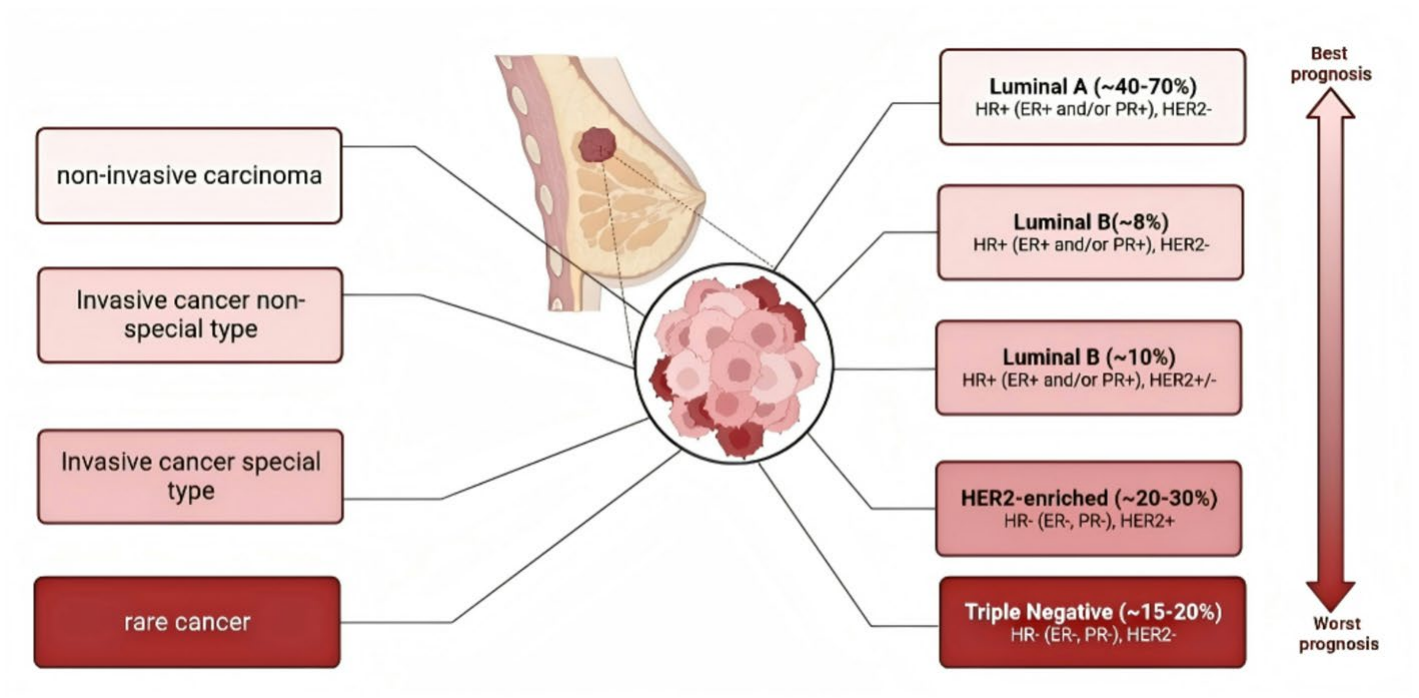
Relationship between pathological types and molecular types of breast cancer and prognosis

Normal mammary tissue undergoes dynamic changes in cell proliferation and apoptosis throughout the physiological cycle, including the processes of proliferation, differentiation, apoptosis and regeneration, all of which are regulated by hormone balance and the processes of proliferation, differentiation, apoptosis and regeneration, all of which [1, 2]. At each stage of mammary gland development, an imbalance between excessive proliferation and abnormal apoptosis of mammary gland cells can lead to the occurrence and progression of tumors [3]. Breast cancer, a malignant tumor originating from breast epithelial cells, is a heterogeneous disease characterized by diverse histological grades and pathological types, exhibiting varying biological behaviors, clinical features, imaging manifestations and prognosis [4, 5]. The common pathological types of breast cancer include ductal carcinoma in situ (DCIS), intraductal carcinoma, lobular carcinoma in situ (LCIS), and Paget’s disease of the nipple. Invasive carcinomas are classified into nonspecial types, such as invasive ductal carcinoma (IDC) and invasive lobular carcinoma (ILC), and special types, including small ductal carcinoma, mucinous carcinoma, and medullary carcinoma, among others [5–8]. In hematoxylin-eosin-stained breast tumor tissue sections, histological grade is evaluated on the basis of the degree of mammary tubule or gland formation, nuclear pleomorphism, and mitotic count, and are categorized into grades I-III, which is an important prognosticfactor for patients with breast cancer [9]. Furthermore, on the basis of the expression of four biomarkers, namely, estrogen receptor (ER), progesterone receptor (PR), human epidermal growth factor receptor 2 (HER2), and ki-67, on the surface of breast cancer cells, breast cancer can be classified into four subtypes: Luminal A, Luminal B, HER2-positive and triple-negative [4]. Some researchers believe that breast cancer originates from different types of stem or progenitor cells, such as luminal carcinoma and basal-like carcinoma, which originate from luminal cells and basal cells, respectively. Luminal-type breast cancers generally have a better prognosis than HER2-positive types do, and triple-negative breast cancers have the worst prognosis [6, 10] (Fig. 1). Consequently, the treatment of breast cancer is characterized by diversity and individualization [11].

**Fig. 2:**
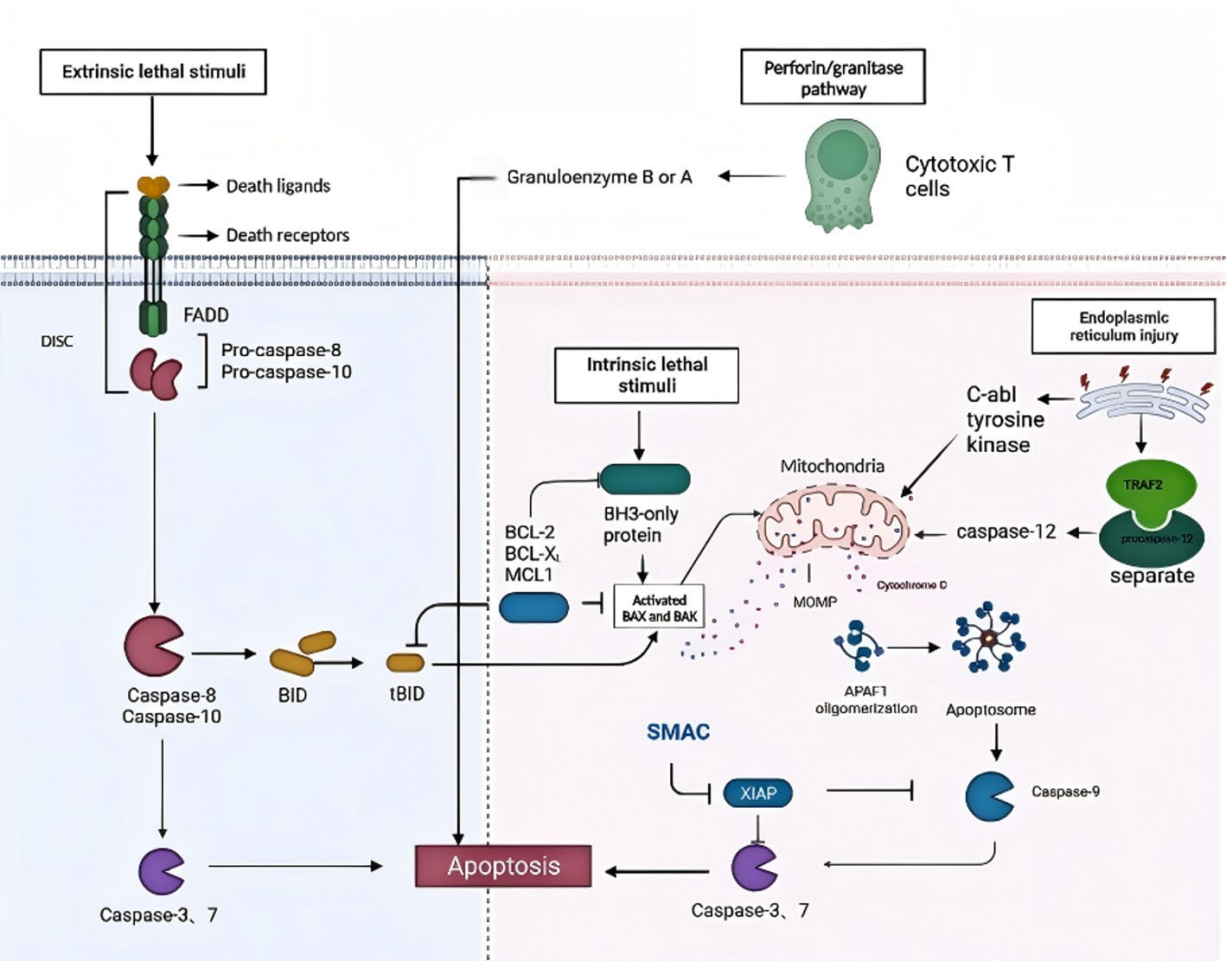
Apoptotic pathways.The extrinsic pathway, Triggered by extracellular ligands (e.g., FasL, TNF-α) binding to transmembrane death receptors (e.g., Fas, TNFR), forming the death-inducing signaling complex (DISC). DISC recruits adaptor protein FADD and initiator procaspase-8/-10, activating caspase-8/-10, which cleaves downstream effector caspases (e.g., caspase-3/-7) to execute apoptosis. The intrinsic pathway, activated by intracellular stressors (e.g., toxins, hypoxia, DNA damage), causing mitochondrial outer membrane permeabilization (MOMP). Cyt c is released into the cytosol, binds apoptotic protease-activating factor-1 (APAF1), and forms the apoptosome with dATP. The apoptosome activates caspase-9, which in turn cleaves effector caspases. Bax/Bak oligomerization: Pro-apoptotic proteins Bax and Bak are activated by caspase-8/-10-mediated cleavage of Bid (tBid) or p53-induced BH3-only proteins, further promoting MOMP. The third pathway is endoplasmic reticulum pathway or ER pathway, ER stress (e.g., hypoxia, calcium imbalance) disrupts ER homeostasis, leading to dissociation of TRAF2 from procaspase-12 and activation of caspase-12. Caspase-12 may stimulate cyt c release, linking the ER pathway to the intrinsic pathway. ER stress also activates c-Abl tyrosine kinase, promoting cyt c release. There is also a perforin/granzyme pathway, in which Cytotoxic T cells release granzyme B/A via perforin, directly activating caspases or inducing mitochondrial damage [16, 17, 29]. (Figure was created with BioRender.com)

Apoptosis, or programmed cell death, is a genetically regulated process of cell elimination. It plays a crucial role in healthy human development and life activities, encompassing processes such as development, differentiation, proliferation homeostasis, immune system regulation, removal of harmful cells, and maintenance of tissue cell population homeostasis in various organs [12, 13]. Dysregulation of apoptosis leads to the development of diseases; for example, increased apoptosis can cause neurodegenerative diseases, AIDS, and ischemic diseases, whereas impaired apoptosis can lead to malignant cell proliferation, such as cancer and autoimmune diseases, which are the core causes of tumor development [12, 14, 15]. The mechanism of apoptosis is complex and includes exogenous pathways (mediated by death receptors), endogenous pathways (mediated by mitochondria), endoplasmic reticulum pathways and perforin/granzyme pathways. These pathways eventually converge on the same executive pathway and are interrelated [16–18](Fig. 2). However, cytochrome c (cyt c) is the core protein in the mitochondrial pathway. Studies have shown that the response of tumors to treatments such as chemotherapy, radiotherapy, and endocrine therapy is, to some extent, achieved through the intrinsic pathway of inducing the release of cyt c and triggering apoptosis. Given its role, cyt c has potential value in cancer treatment [18, 19].

The purpose of this review is to explore the function and mechanism of cyt c-mediated apoptosis in the occurrence and treatment of breast cancer, indicating that cytochrome c plays an important role in the occurrence and treatment of breast cancer, emphasizing the critical role of cyt c in this process. By elucidating the involved mechanisms, we aim to provide evidence for relevant research ideas and detection methods, thereby facilitating the discovery of new directions for future breast cancer diagnosis and treatment strategies.

## Molecular mechanisms of cyt c in apoptosis

cyt c, derived from somatic cells, is a water-soluble 13 kDa hemoglobin-containing protein encoded by nuclear genes [20]. It serves as a pivotal component of the mitochondrial respiratory electron transport chain and acts as an indispensable activator of programmed cell death or apoptosis [21]. When cyt c is normally localized within the cristae of the inner mitochondrial membrane, it undergoes a critical transition when intrinsic apoptotic pathways are activated. These pathways can be triggered by various stimuli, including DNA damage, metabolic stress, or the accumulation of unfolded proteins. Upon activation, cyt c is released from the mitochondrial intermembrane space into the cytoplasm [22]. In the presence of dATP, it binds to cytoplasmic apoptotic protease-activating factor-1 (Apaf-1), forming an oligomeric complex known as the apoptosome. The core of this complex consists of seven symmetrically arranged APAF1 molecules, forming a wheel-like structure. This structure serves as a signaling platform that facilitates the autoactivation of the initiator caspase, caspase-9. Once activated, caspase-9 undergoes cleavage to form its catalytically active form, which in turn activates the downstream effector caspases, caspase-3 and − 7. These effector caspases orchestrate a series of cellular events that culminate in cell death. Notably, in the absence of cyt c, Apaf-1 fails to activate caspase-9, highlighting the critical role of cyt c in this apoptotic cascade. In addition, the release of cyt c disrupts the electron transport chain, leading to ATP depletion and exacerbating cell death [22–25].

## The role of cyt c in breast cancer

The normal development and function of the breast depend on the balance between cell proliferation and cell apoptosis [26]. The traditional genetic view holds that tumors are caused by the abnormal activation of oncogenes or the abnormal inactivation of tumor suppressor genes [27]. An increasing number of studies have shown that impaired apoptosis can also lead to tumor development, which may be due to the transformation of oncogenes or tumor suppressor genes, where apoptosis is protected, cells fail to receive apoptotic signals, and the apoptotic pathway is damaged, thus leading to malignant proliferation [18, 28]. The mitochondrial-related apoptotic pathway is considered an important factor in the occurrence of cancer and can lead to the development of various cancers [29]. Studies have shown that in breast cancer tumor samples, cyt c is released from epithelial cells into the cavity of the cancerous duct, accompanied by a cyt c redox imbalance, where reduced cyt c cannot induce apoptosis and is upregulated at all stages of cancer development [19]. Some gene regulatory mechanisms lead to a reduction in the expression or release of cyt c, resulting in insufficient cell apoptosis, which is associated with the lower survival rate of patients [29–31]. Some proteins within cells competitively bind to Cyt c, inhibiting its interaction with Apaf-1, thereby protecting breast cancer cells from apoptosis [32]. In patients with triple-negative breast cancer (TNBC), small extracellular vesicles (sEVs) in the plasma, especially exosomes derived from tumor cells (TEXs), when they enter T cells, induce the release of cyt c and Smac from the mitochondria and activate the cleavage of caspase-3 and PARP in the cytoplasm, thereby promoting T-cell dysfunction and leading to tumor progression [33]. Some studies have indirectly shown that a decrease in cyt c is related to patient resistance [31, 34–36]. In addition to chemotherapy, systemic treatment also includes endocrine therapy, anti-HER2 targeted therapy and immunotherapy for different molecular types of breast cancer. Although the mechanisms are different, they ultimately promote the expression and release of cyt c and increase the sensitivity of tumor cells to apoptosis [15, 37, 38]. Delivering exogenous cyt c into the cytoplasm of cancer cells is an effective method for inducing the apoptosis of cancer cells [29]. These findings highlight the crucial role of cyt c in the occurrence, development and treatment of breast cancer.

## Potential of cyt c as a therapeutic target in breast cancer

**Fig. 3:**
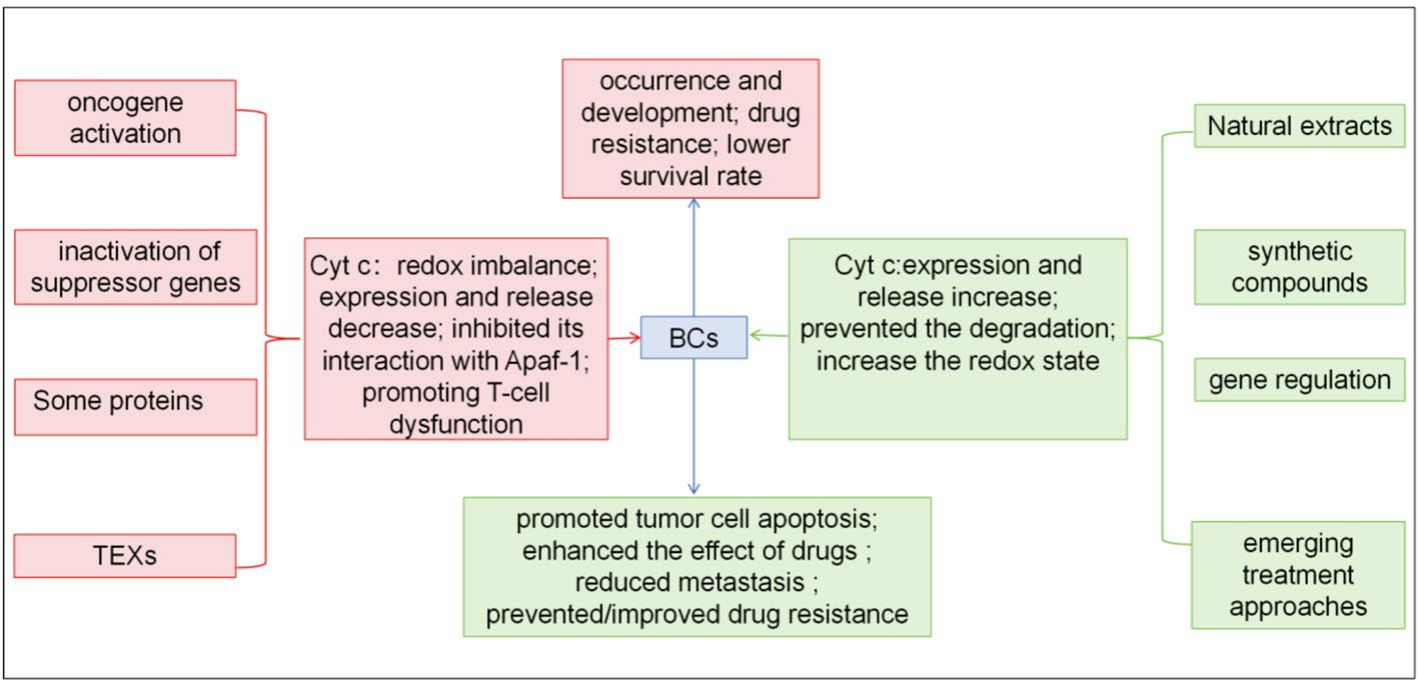
A schematic diagram illustrating the role of cytochrome c in the onset and treatment of breast cancer

An increasing number of studies have shown that natural extracts, synthetic compounds, gene regulation methods, and emerging treatment approaches can effectively induce apoptosis in breast cancer cells by promoting the expression or release of cyt c, demonstrating significant anticancer potential. The relevant research progress can be summarized as follows (Fig. 3).

### Natural extracts promote apoptosis by regulating cyt c

Numerous natural products derived from plants have been shown to effectively trigger the mitochondrial apoptosis pathway in breast cancer cells, as summarized in Table 1. Achillea fragrantissima, a shrub plant belonging to the Asteraceae family native to Arabia and Egypt, significantly upregulates the expression of apoptosis-related genes, including caspase-3, caspase-9, cyt c, BID, BAX, and PTEN, while concurrently downregulating the PI3K/Akt signaling pathway. This plant extract exerts a potent anticancer effect on MCF-7 cells by inducing apoptosis [39]. Moringa, an isothiocyanate isolated from the seeds of Moringa oleifera, interacts with PHB, dynamic associated protein 1 (DRP1), and mitochondrial localization protein 2 (SLP2), leading to the induction of proapoptotic proteins such as cyt c, p53, and cleaved caspase-7. It induces apoptosis through the mitochondrial pathway, effectively inhibiting the growth of MCF-7 and MDA-MB-231 cells [40]. Apigenin, a flavone commonly found in onion, grapefruit, orange, parsley, and chamomile, activates intrinsic apoptotic pathways by inducing cyt c, Bax, and caspase 3. It exhibits significant antitumor activity in various tumor types, including breast cancer and lung cancer [41]. Catalpol, an iridoid glycoside extracted from Rehmannia glutinosa, a traditional Chinese medicinal plant, causes the loss of mitochondrial membrane potential (MMP), increases in reactive oxygen species (ROS) production and elevates cytoplasmic cyt c levels in MCF-7 cells. By regulating the mitochondrial apoptotic pathway, it exerts an inhibitory effect on breast cancer both in vitro and in vivo [42]. CREE, derived from the root of Cimicifuga dahurica (Turcz.) Maxim (C. dahurica), upregulates the expression levels of Bax, caspase-9/3, and cyt c, thereby inducing apoptosis via the mitochondrial pathway and significantly inhibiting the proliferation, migration, and invasion of human breast cancer MCF-7 and MDA-MB-231 cells [43]. Diallyl trisulfide (DATS), a bioactive compound from Allium vegetables, regulates cyt c levels, exerting cancer-fighting potential by inducing apoptosis in micro mammary DCIS and invasive breast cancer cells [44]. Oleuropein, the predominant polyphenol component of olive fruits and leaves, promotes apoptosis in MDA-MB-468 cells by inducing the expression of the cyt c gene [45]. Plumbagin (5-hydroxy-2-methyl-1,4-naphthoquinone), initially isolated from the roots of Plumbago zeylanica, P. rasea, and P. europoea and Nepenthes plants, significantly increases the Bax/Bcl-2 ratio and promotes cyt c release, thereby inducing apoptosis in MCF-7 cells [46]. Sophoraflavanone G (SG), which is isolated from Sophora flavescens, inhibits MDA-MB-231 cells by reducing the expression of the antiapoptotic proteins Bcl-2 and Bcl-xL while increasing the expression of the proapoptotic protein Bax. It also facilitates the release of cyt c from mitochondria into the cytoplasm, enhancing apoptosis and reducing the migration and invasion abilities of breast cancer cells [47].

**Table 1:** The information of the natural extracts

Name	Source	References
Achillea fragrantissima	is a shrub plant that belongs to the Asteraceae family in Arabia and Egypt	[39]
Moringin	an isothiocyanate derived from the seeds of Moringa oleifera	[40]
Apigenin	is a flavone commonly present in onions, grapefruit, oranges, parsley, and chamomile	[41]
Catalpol	an iridoid glycoside extracted from the traditional Chinese medicinal plant Rehmannia glutinosa	[42]
CREE	extract of the root of C. dahurica	[43]
DATS	a bioactive compound from Allium vegetables	[44]
Oleuropein	the most prominent polyphenol component of olive fruits and leaves	[45]
Plumbagin	was isolated from the roots of Plumbago zeylanica, P. rasea, and P. europoea and Nepenthes plants	[46]
Sophoraflavanone G	Sophora flavescens	[47]

### Compounds or their combination strategies can induce cell apoptosis by influencing cyt c

Research has shown that certain synthetic compounds, when combined with other drugs or targeting specific targets, can more effectively induce mitochondrial apoptosis(Table 2). Isobutyl-deoxynyboquinone (IB-DNQ) is a potent and specific NQO1-bioactivatable futile redox cycling molecule that, combined with the inhibition of superoxide dismutase 1 (SOD1), can specifically increase the killing effect on tumor stem cells (CSCs) and induce mitochondrial oxidative damage, cyt c release, and activation of the caspase-3-mediated apoptotic pathway, effectively inhibiting the growth, metastasis and tumor-initiating potential of TNBC [48]. ZK-CH-11d is a synthetic indole chalcone derivative that promotes cyt c release, increases the activity of caspase 3 and caspase 7, and induces apoptosis. It has a significant antiproliferative effect on breast cancer cells and has a relatively minor effect on noncancer cells. The study also found that ZK-CH-11d can activate autophagy. However, chloroquine (an autophagy inhibitor) significantly enhanced the cytotoxicity of ZK-CH-11d in MDA-MB-231 cells, indicating that autophagy is not the main mechanism of ZK-CH-11d’s anti-proliferative effect. Based on the combined experimental results, it is speculated that autophagy is activated as a defense mechanism of the cells [49]. Tricyclohexylphosphine gold (I) n-mercaptobenzoate complexes promote the generation of ROS in MCF-7 cells, the release of cyt c, and the activation of caspases 3/7, 8, 9, and 10, exerting an antitumor effect by inducing apoptosis. Furthermore, the cytotoxicity of this compound is superior to that of the positive control drug cisplatin [50]. Other studies have shown that when chloroquine is combined with isorhamnetin (IH), it can induce mitochondrial division, which is accompanied by the translocation of Bax to the mitochondria and the release of cyt c, which induces the apoptosis of TNBC cells and enhances the efficacy of chemotherapeutic drugs [51]. Chetomin (CHET) is a fungal metabolite synthesized by Chaetomium cochloides and causes calcium overload in mitochondria and mitochondrial dysfunction, leading to the release of cyt c, thereby triggering caspase-3-mediated cell death and inhibiting the growth of TNBC cells [52]. Bortezomib is a proteasome inhibitor that can restore the expression of apoptotic proteins such as Apaf-1 and prevent the degradation of cytosolic cyt c released by Docetaxel, thereby triggering the intrinsic apoptotic pathway and promoting cancer cell death, enhancing the cytotoxic effect of docetaxel on MCF7 breast cancer cells, and it also helps to overcome chemoresistance [53]. S2E is a glutathione S-transferase omega 1 (GSTO1) inhibitor. When used in combination with tamoxifen, it activates the c-Jun N-terminal kinase stress kinase, inducing the mitochondrial apoptotic signaling pathway through the proapoptotic proteins BAX and cyt c in breast cancer CSCs, thereby increasing breast cancer cell apoptosis and reducing their migration and invasion abilities [54]. Indole-2-carboxamides are EGFR/CDK2 dual inhibitors that induce cell apoptosis with high efficiency by influencing apoptotic markers (such as caspases 3, 8, and 9; cyt c; Bax; Bcl2; and p53). Compared with the control drug doxorubicin, it has significant anti-proliferative effects on MCF-7 cells [55]. Pt12 is a novel berenil complex of platinum(II) that, when used in combination with a monoclonal antibody against MUC1, increases the concentrations of proapoptotic Bax, cyt c, and caspase-9, strongly inducing the apoptosis of MCF-7 cells through both external and internal apoptotic pathways and resulting in stronger anticancer effects [56]. MMD is a new quinazolinone compound that can increase the release of cyt c from mitochondria to the cytoplasm, and this effect increases over time. It promotes the apoptosis of MCF-7 cells through the intrinsic mitochondrial pathway [57]. Cinnarizine is a potential drug that targets protein kinase C (PKC), interferes with the cell cycle of breast cancer cells (reducing S-phase cells and increasing G1-phase cell accumulation), damages the mitochondrial membrane potential, releases cyt c into the cytoplasm, and activates caspase-3, which inhibits the growth of breast cancer cells by inducing apoptosis [58]. The aryl-ureido fatty acid CTU is a prototype of a new class of targeted agents that are capable of targeting mitochondria, depolarizing the mitochondrial membrane, activating ROS production, and destroying the IMM and OMM, thereby releasing cyt c and promoting the death of MDA-MB-231 cells [59]. Paclitaxel is used in combination with sorafenib and radiotherapy and has a significant anticancer effect on breast cancer. In particular, the induction of mitochondrial cyt c dependent apoptosis can more effectively inhibit tumor growth and can improve the survival rate and quality of life of patients [60]. etinoic acid and retinol can regulate the redox state of cyt c in the electron transport chain that controls oxidative phosphorylation and apoptosis, which is crucial for mitochondrial energy homeostasis. They also promote the release of cyt c from the mitochondria, thereby facilitating apoptosis [61].

**Table 2:** The compounds and their mechanism of affecting cyt c

Compounds	Combination/Targets	effect	References
IB-DNQ	Combined with the inhibition of SOD1	Inducing the release of cyt c leads to apoptosis in MCF-7 cells	[48]
ZK-CH-11d	Combined with Chloroquine	Promotes cyt c release, enhanced the cytotoxicity of ZK-CH-11d in MDA-MB-231 cells	[49]
Tricyclohexylphosphine gold (I) n-mercaptobenzoate complexes	Inhibit TrxR	Promotes cyt c release, leads to apoptosis in MCF-7 cells to exert anti-tumor effects	[50]
Chloroquine	Combined with Isorhamnetin	Inducing mitochondrial division, and the release of cyt c, leads to apoptosis in TNBC cells and enhance the efficacy of chemotherapy drugs	[51]
Chetomin(CHET)	Increase the level of Ca2 + in mitochondria	Leading to the release of cyt c, and inhibits the growth of TNBC cells	[52]
Bortezomib	Docetaxel	Prevent the degradation of cytosolic cyt c, enhance the cytotoxic effect of docetaxel on MCF7 breast cancer cells and possibly overcome chemotherapy resistance	[53]
S2E	Combined with tamoxifen	Inducing cyt c in breast cancer CSCs, Preventing breast cancer metastasis and recurrence mediated	[54]
Indole-2-Carboxamides	Inhibit EGFR and CDK2	induce cell apoptosis by influencing cyt c, compared with doxorubicin, it exhibited significant anti-proliferative activity against MCF-7 cells.	[55]
Pt12	Combined with monoclonal antibody against MUC1	increases the concentrations of cyt c, induces apoptosis in MCF-7 breast cancer cells	[56]
MMD	/	increases the release of cyt c, promotes apoptosis of breast cancer cells	[57]
Cinnarizine	targets PKC	damages the mitochondrial membrane potential, releases cyt c into the cytoplasm, inhibit the growth of breast cancer cells by inducing cell apoptosis	[58]
The aryl-ureido fatty acid CTU	Targets mitochondria	releasing cyt c and promoting the death of MDA-MB-231 cells	[59]
Paclitaxel:	Combined with sorafenib and radiotherapy	by inducing mitochondrial cyt c dependent apoptosis, it can more effectively inhibit tumor growth and may improve the survival rate and quality of life of patients	[60]
Retinoids	Combined with Retinol	Alter the redox state of cyt c and promote the apoptosis of breast cancer cells	[61]

### The gene and signaling pathway regulation cyt c

Alterations in key genes and signaling pathways within cells directly affect the sensitivity of the mitochondrial apoptotic pathway (Table 3). Such as, by activating PP1, wild-type p53 increases the level of cyt c and promotes the apoptosis of breast cancer cells [62]. RAP80 is a member of the BRCA1-A complex. In the cell nucleus, it plays an important role in regulating cell cycle checkpoints and DNA damage repair. It participates in the repair of DNA damage induced by cisplatin. Inhibiting RAP80 expression can upregulate the protein expression of Caspase-3, Apaf-1, cyt c, Bax and Fas; induce the apoptosis of breast cancer cells; and increase their chemosensitivity to cisplatin [63]. The interaction of SH3GL2 (a protein related to vesicle endocytosis) and MFN2 (an important regulator of mitochondrial fusion) in mitochondria induces the production of superoxide anions and the release of cyt c from mitochondria to the cytoplasm, which can significantly reduce the metastasis of breast cancer to the lungs and liver and slow the growth of the primary tumor [64]. miR-24-2 downregulates the expression of Akt, Erk1/2, Bcl-2 and mitochondrial cyt c and increases cyt c in the cytoplasm, promoting apoptosis and thereby inhibiting the growth and progression of TNBC [65]. In MDA-MB-231 breast cancer cell lines resistant to cisplatin or doxorubicin, inhibiting hematopoietic cell-specific protein 1-associated protein X-1 (HAX-1) can induce the release of cyt c from mitochondria, which helps restore the sensitivity of breast cancer cells to chemotherapy drugs [34]. Inhibiting ADP-ribosylation factor 1 (ARF1) inhibits the activation of key survival mediators (such as ERK1/2, AKT and Src) mediated by gefitinib while enhancing apoptotic signaling pathways (such as the p38MAPK and JNK pathways) and changing the ratio of Bax/Bcl2 and the release of cyt c, improving the resistance of triple-negative breast cancer to EGFR inhibitors and enhancing the therapeutic effect of EGFR inhibitors [66]. Macrophage migration inhibitory factor (MIF) is a pleiotropic proinflammatory cytokine that is abnormally expressed in various solid tumors and is known to promote tumor progression and metastasis. The inhibitor of MIF, CPSI-1306, induces intrinsic apoptosis by altering the mitochondrial membrane potential, releasing cyt c, and activating different caspases, thus inhibiting the progression and metastasis of TNBC [67]. The ER-positive breast cancer cell line (MCF-7:5 C) is resistant to long-term estrogen deprivation. However, compared with cells treated with fulvestrant or the vehicle, MCF-7:5 C cells treated with estradiol underwent apoptosis and exhibited increased expression of proapoptotic proteins and increased release of cyt c. And physiological concentrations of estradiol may be used to induce apoptosis and tumor regression in tumors that develop resistance to aromatase inhibitors [35].

**Table 3:** The gene and signaling pathway regulation cyt c

gene/signaling pathway	Ctivation/Inhibit	effect	References
Wild-type p53	Activates PP1	Increases the level of cyt c, promoting apoptosis of breast cancer cells	[62]
RAP80	Inhibits RAP80	Induces the expression of cyt c, promotes apoptosis of breast cancer cells, and enhances their chemosensitivity to cisplatin	[63]
SH3GL2	Interacts with MFN2	Induces the production of superoxide anion and the release of cyt c, significantly reducing the metastasis of breast cancer to the lungs and liver, and slowing the growth of the primary tumor	[64]
miR-24-2	Overexpression of miR-24-2	Increases the expression and release of cyt c, promotes apoptosis, thereby inhibiting the growth and progression of TNBC	[65]
HAX-1	Inhibits HAX-1	Can induce the release of cyt c from mitochondria, helping to restore the sensitivity of breast cancer cells to chemotherapy drugs	[34]
ARF1	Inhibits ARF1	Promotes the release of cyt c, improving the resistance of triple-negative breast cancer to EGFR inhibitors	[66]
MIF	Inhibitor CPSI-1306	Induces intrinsic apoptosis by altering mitochondrial membrane potential, releasing cyt c, and activating different caspases, inhibiting the progression and metastasis of TNBC	[67]
Estrogen receptor (ER)	Estradiol	Increases release of cyt c, and used for tumors resistant to aromatase inhibitors to promote apoptosis and tumor regression	[35]

### Some emerging treatment methods induce apoptosis by regulating cyt c

In recent years, breakthroughs have been continuously made in the field of medicine, and some new treatment methods or combined treatment plans have emerged one after another. These innovative therapies induce apoptosis by promoting the release of cyt c, providing new hope for improving the therapeutic effect on breast cancer (Table 4). Taking HER2-positive breast cancer as an example, researchers have developed a new treatment strategy. This strategy ingeniously combines dual receptor-targeted lipid-encapsulated oxygen nanobubbles (DRT@Lipo-NBs-O₂), photodynamic therapy (PDT), and immune checkpoint inhibitors (ICIs). This combined therapy can improve the tumor microenvironment, increasing sensitivity to immune checkpoint inhibitors, promoting cyt c induced tumor cell death and significantly enhancing the therapeutic effect on HER2-positive breast cancer [68]. There have also been remarkable research achievements in the treatment of lung metastasis in patients with breast cancer. A mitochondrial-targeted doxorubicin delivery system based on an N-(2-hydroxypropyl) methylacrylamide copolymer combined with the mitochondrial-distributed Bcl-2 functional transformation peptide NuBCP-9 delivery system has demonstrated strong antimetastatic ability. This combined therapy successfully reduced the lung metastasis rate of breast cancer patients by 84% by increasing the expression of cyt c and simultaneously reducing the expression of Bcl-2, matrix metalloproteinase-9 (MMP-9), and vascular endothelial growth factor(VEGF) [69]. Curcumin TPP-PEG-PCL nanomicelles also perform well in the treatment of breast cancer. It can promote the uptake of drugs by tumor cells, escape the phagocytosis of lysosomes, and precisely target mitochondria. During the course of action, these nanomicelles significantly reduce the mitochondrial membrane potential of breast cancer cells, increase the release of cyt c, simultaneously increase the expression of the proapoptotic protein Bax, and reduce the expression of the antiapoptotic protein Bcl-2, thereby enhancing the ability of the drug to promote the apoptosis of tumor cells [70]. Fenton-reaction-stimulated nanoparticles (P@P/H NPs) are also highly promising nanomedicines. It can significantly increase the level of reactive oxygen species (ROS). In in vitro experiments, by promoting the expression of cyt c, caspase-9 and caspase-3 and blocking the expression of matrix metalloproteinase 9 (MMP-9), it has a significant antimetastatic effect on triple-negative breast cancer (TNBC) [71]. For triple-negative breast cancer (TNBC), combined treatment with autophagy inhibitors and the EGFR inhibitor gefitinib has also made positive progress. This combined therapy can increase the BAX/Bcl-2 ratio, increase the release of cyt c, and increase the level of caspase-3(CASP3), thereby activating the mitochondrial dependent apoptotic pathway, promoting apoptosis, and significantly improving the therapeutic effect of EGFR inhibitors [72]. In the treatment of estrogen receptor (ER)-positive breast cancer, the combination of HDAC inhibitors and antiestrogen drugs also has unique advantages. This combined therapy can promote cyt c dependent apoptosis, but some cells develop resistance to apoptosis under combined therapy and exhibit strong autophagy induction. Moreover, the addition of autophagy inhibitors can further promote the induction of apoptosis, enhance the therapeutic effect of antiestrogen drugs on ER-positive breast cancer, and may also help prevent the development of drug resistance [73]. These emerging treatment methods and combined treatment regimens have opened new paths for the treatment of breast cancer and are expected to bring good news to more patients in the future.

**Table 4:** Emerging treatment methods regulating cyt c

Type of treatment	therapeutic regimen	effect	References
Immunotherapy	DRT@Lipo-NBs-O2, PDT and ICIs	Make the tumor microenvironment more sensitive to ICIs, and promote the death of tumor cells induced by cyt c, thereby improving the therapeutic effect of HER2-positive breast cancer.	[68]
Targeted therapy	Mitochondrial-targeted doxorubicin delivery system and Bcl-2 function-converting peptide NuBCP-9 delivery system	By increasing the expression of cyt c, it was successfully achieved to reduce lung metastasis of breast cancer by 84%.	[69]
Nano materials	Curcumin TPP-PEG-PCL nanomicelles	Promote the uptake of drugs by tumor cells, reduce the mitochondrial membrane potential of breast cancer cells, increase the release of cyt c, and enhance the effect of drugs in promoting tumor cell apoptosis	[70]
Nano materials	Fenton-reaction-stimulative nanoparticles (P@P/H NPs)	Significantly increase the level of ROS. In vitro, by promoting the expression of cyt c, it exhibits a significant anti-TNBC metastasis effect.	[71]
Targeted therapy	Autophagy inhibitors and EGFR inhibitors	Activating the mitochondrial-dependent apoptotic pathway and promoting cell apoptosis significantly enhanced the therapeutic effect of EGFR inhibitors.	[72]
Targeted therapy	HDAC inhibitors, anti-estrogen drugs, autophagy inhibitors	Enhancing the efficacy of anti-estrogen drugs in treating ER-positive breast cancer and possibly preventing the development of drug resistance	[73]

## Detection of cyt c in breast cancer cells

Cyt c plays crucial roles in the occurrence, development, and treatment of breast cancer. Therefore, exploring methods that can effectively detect cyt c in breast cancer cells is particularly important. Research indicates that cyt c plays a core role in the process of cell apoptosis. However, its function may be impaired by interactions with antiapoptotic proteins such as HSP27 and Bcl-xL. To further investigate this interaction, a method combining molecularly imprinted polymers (MIPs) with liquid chromatography‒tandem mass spectrometry (LC‒MS‒MS/MS)-targeted proteomics has emerged. This method can simultaneously and quantitatively detect the interaction between cyt c and two antiapoptotic proteins, HSP27 and Bcl-xL [74]. Raman imaging technology provides a unique perspective for the study of breast cancer. Through this technology, researchers can observe the specific locations and distributions of various biochemical components in the lumen, epithelial cells, and extracellular matrix of human breast ductal carcinoma during its development. Compared with normal tissues, breast cancer tissues are in a state of redox imbalance, and reduced cyt c is upregulated at all stages of cancer development [19]. Additionally, this technology, by monitoring the redox state of cyt c, reveals the impact of retinoids substances on human breast cancer [61]. Moreover, some studies have revealed a close relationship between cyt c levels in serum and the apoptotic process: the inhibition of apoptosis is often accompanied by a decrease in cyt c levels in serum, whereas the induction of apoptosis leads to an increase in its levels [75]. These findings indicate that the quantitative analysis of cyt c in serum has great potential value for monitoring patients’ responses to chemotherapy and assessing patient prognosis. To meet this clinical need, researchers have successfully developed various highly sensitive biosensors for the quantitative analysis of cyt c levels in human serum. The emergence of these biosensors provides doctors with powerful tools for evaluating patients’ treatment response and prognosis [29]. In the future, these detection methods can be used to further explore the correlation between the cyt c concentration and the grades of different types of cancer and to evaluate cancer prognosis and the efficacy of anticancer drugs.

## Conclusion

In summary, cyt c plays a crucial role in the pathogenesis and therapeutic of breast cancer. Its function in the mitochondrial apoptotic pathway highlights its importance in regulating cell survival and death, especially in the context of cancer progression and treatment response. This review highlights the multifaceted involvement of cyt c in breast cancer, ranging from its dysregulation in tumor cells to its potential as a therapeutic target. These findings underscore the importance of strategies that promote cyt c release to induce apoptosis in cancer cells, which can be achieved through natural extracts, synthetic compounds, or the regulation of genes and signaling pathways. Additionally, the development of innovative methods for detecting cyt c in breast cancer cells and serum offers promising avenues for improving diagnosis, prognosis assessment, and treatment monitoring. Currently, most research on breast cancer treatments that target cyt c or its related apoptotic pathways remains in the basic research and preclinical stages. As research continues to unravel the complexity of cyt c in cancer biology, more targeted and effective treatment options are expected to emerge, ultimately enhancing the clinical outcomes of patients with breast cancer.

## Data Availability

The datasets generated during and/or analyzed during the current study are available from the corresponding author upon reasonable request.
